# Repurposing Anti-Malaria Phytomedicine Artemisinin as a COVID-19 Drug

**DOI:** 10.3389/fphar.2021.649532

**Published:** 2021-03-19

**Authors:** Fatih M. Uckun, Saran Saund, Hitesh Windlass, Vuong Trieu

**Affiliations:** ^1^ Ares Pharmaceuticals, LLC, St. Paul, MN, United States; ^2^ Oncotelic Inc., Agoura Hills, CA, United States; ^3^ Windlas Biotech Pvt. Ltd., Dehradun, India

**Keywords:** COVID-19, ARDS, TGF — Transforming growth factor, malaria, anti-inflammatory

## Abstract

Artemisinin is an anti-inflammatory phytomedicine with broad-spectrum antiviral activity. Artemisinin and its antimalarial properties were discovered by the Chinese scientist Tu Youyu, who became one of the laureates of the 2015 Nobel Prize in Physiology or Medicine for this breakthrough in tropical medicine. It is a commonly used anti-malaria drug. Artemisinin has recently been repurposed as a potential COVID-19 drug. Its documented anti-SARS-CoV-2 activity has been attributed to its ability to inhibit spike-protein mediated and TGF-β-dependent early steps in the infection process as well as its ability to disrupt the post-entry intracellular events of the SARS-CoV-2 infection cycle required for viral replication. In addition, Artemisinin has anti-inflammatory activity and reduces the systemic levels of inflammatory cytokines that contribute to cytokine storm and inflammatory organ injury in high-risk COVID-19 patients. We postulate that Artemisinin may prevent the worsening of the health condition of patients with mild-moderate COVID-19 when administered early in the course of their disease.

## Introduction

New effective drugs are needed to prevent the potentially deadly complications of COVID-19 (Woolf et al., 2020; Faust et al., 2021; Woolf et al., 2021) and thereby reduce its fatality rate (Uckun, 2020a; Uckun, 2020b; Uckun et al., 2020a; Zheng et al., 2020; Zhou et al., 2020). The goal of this mini-review is to discuss the emerging evidence regarding the clinical potential of Artemisinin for the treatment of COVID-19.

Due to their favorable safety profiles, natural products and phytomedicines are being explored as potential therapeutic or prophylactic agents with different mechanisms of action against COVID-19 (Huang et al., 2020). Some natural products have the potential to impair the attachment of SARS-CoV-2 spike glycoprotein to its receptors on human cells, including the Heat Shock Protein A5 (HSPA5) substrate-binding domain β (SBDβ) and angiotensin-converting enzyme 2 (ACE2) receptor (Elfiky, 2020; Kumar et al., 2020). Others have been proposed as inhibitors of viral replication, such as the recently reported compounds derived from Alpinia officinarum and ginger that may affect SARS-CoV-2 replication by blocking the SARS-CoV-2 papain-like protease (PLpro) (Goswami et al., 2020), compounds derived from African plants that may inhibit the 3-chymotrypsin-like protease (3CL ^pro^): (Gyebi et al., 2020), or natural polyphenols such as quercetin that may inhibit the RNA-dependent RNA polymerase (RdRp) (El-Aziz Abd et al., 2020). In addition, several natural products have immunomodulatory activities that may have clinically beneficial anti-inflammatory effects, including Chinese herb prescriptions Huang et al., 2020; Xu and Zhang, 2020).

*Artemisia* species contain bioactive substances with pleiotropic biological effects (Li et al., 2018). For example, *Artemisia annua* contains anti-inflammatory sesquiterpenoids, including Artemisinin (viz.: artesunate). Artemisinin and its antimalarial properties were discovered by the Chinese scientist Tu Youyu, who became one of the laureates of the 2015 Nobel Prize in Physiology or Medicine for this discovery (Li et al., 2018).

Artemisinin and some of its derivatives exhibit *in vitro* antiviral activity against a number of pathogenic human viruses, such as human cytomegalovirus (HCMV), Epstein Barr virus (EBV), human herpes simplex virus-6 (HHV-6) (Efferth et al., 2008; D’alessandro et al., 2020). Case reports of clinical response have been reported in a child with HHV-6 myocarditis and a patient with ganciclovir-resistant, foscarnet-resistant HCMV (Efferth et al., 2008; D’alessandro et al., 2020). *In vivo* antiviral activity was observed in the rat CMV model and a murine model of herpes simplex encephalitis (HSE) as well (Efferth et al., 2008). Studies by Cao et al. (2020) and Gilmore et al. (2020) confirmed the antiviral activity of Artemisinin and its derivatives against SARS-2-CoV-2 at micromolar concentrations. Recent docking studies indicated that Artemisinin and its derivative Artesunate could bind the SARS-CoV-2 spike protein in a way that would interfere with its docking onto the human ACE2 receptor protein, which is the required first step in the host infection process of the coronavirus disease 2019 (COVID-19) (Sehailia and Chemat, 2020; Yan et al., 2020). Importantly, recent research by Cao et al. revealed that Artemisinin-related compounds Arteannuin B and Lumefantrine disrupted the post-entry intracellular events of the SARS-CoV-2 infection cycle required for viral replication (Cao et al., 2020). Therefore, when these artemisinins were added at clinically achievable micromolar concentrations throughout the infection process or post-entry (but not when added before or during virus entry), SARS-CoV-2 replication was effectively inhibited, as measured by quantitative RT-PCR or viral RNA and protein assays (Cao et al., 2020).

### Clinical Safety Profile and Pharmacokinetics of Artemisinin

Orally administered Artemisinin and Artemisinin derivatives are generally well-tolerated, especially when used for a short treatment course (Duc et al., 1994; De Vries et al., 1997; Ashton et al., 1998; Gordi et al., 2002; Hien et al., 2011; Li et al., 2018; Wang et al., 2020; Li et al., 2021). Except for the rare occurrence of hepatotoxicity and mild-moderate headache, nausea, vomiting, fatigue, and anorexia, Artemisinin was found to be clinically safe in healthy volunteers as well as malaria patients (Duc et al., 1994; De Vries et al., 1997; Ashton et al., 1998; Gordi et al., 2002; Hien et al., 2011; Li et al., 2018; Wang et al., 2020; Li et al., 2021). Severe hemolytic anemia requiring transfusion is a well-documented complication encountered within 28 days of therapy initiation by 20–25% of malaria patients treated with parenterally administered Artusenate and it necessitates close clinical monitoring for risk mitigation (Jauréguiberry et al., 2014; Savargaonkar et al., 2020). Likewise, severe hemolytic anemia requiring blood transfusions after oral artemisinin therapy has been observed as a rare complication in malaria patients with high parasite loads (Conlon et al., 2020). Based on its overall favorable safety profile, the World Health Organization (WHO) recommends parenteral artesunate for the treatment of severe malaria (WHO) (WHO, 2010).

### Clinical Activity of Artemisinin and Chemical Derivatives of Artemisinin in COVID-19 Patients

Some clinical trials also suggested that Artemisinin may contribute to a faster recovery of COVID-19. Li et al. reported the results from an open-label non-randomized study in which 41 COVID-19 patients received either standard of care (SOC) therapy (control) or SOC combined with Artemisinin plus piperaquine (AP) (Li et al., 2021). The average time to reach undetectable viral RNA was significantly shorter for the AP group (Li et al., 2021). Patients in the AP group showed a faster clearance of SARS-CoV-2 than control patients. Liver enzyme elevations, as well as QTc interval prolongations on ECGs were observed in the AP arm, consistent with hepatotoxicity and cardiac toxicity.

ArtemiC is a medical spray containing Artemisinin, curcumin, Frankincense resin from the Boswellia sacra tree and Vitamin C. In the controlled Phase II trial NCT04382040, patients with COVID-19 received ArtemiC spray in addition to standard care. Study data have not been published in a peer-review article, but a press release of the preliminary data suggested that ArtemiC may be more active than placebo in contributing to the improvement of the patients’ condition (Health Care, 2020). Likewise, the efficacy signal for the Artemisinin derivative Artesunate during a recently completed prospective, controlled clinical COVID-19 study was promising. In Artesunate treatment group, time to significant improvement of the symptoms, time to conversion to negativity of SARS-CoV-2 tests, and length of hospital stay was shorter than in the control group (Lin et al., 2020).

There are several Phase II/III studies currently underway in which pharmaceutical compositions or supplements containing Artemisinin and/or its derivatives are being evaluated as adjuncts to the standard of care in COVID-19 patients, including but not limited to Artesunate plus Artemisinin (NCT04387240), Artesunate plus amodiaquine (NCT04502342); Artesunate plus pyronaridine (NCT04475107), Artusunate as well as Artemisia annua (NCT04374019). In the CTRI/2020/09/028044 randomized Phase 4 trial, the efficacy of ARTIVeda (Artemisinin) is being studied in COVID-19 patients with mild-moderate disease. The product, ArtiVeda™ (License # UK.AY-401/2018, Ministry of AYUSH, India), is a novel gelatin capsule formulation of the Artemisia extract Ayurveda for oral delivery of the active ingredient Artemisinin for treatment of COVID-19. Pending the comparative evaluation of the pending data, it would be helpful to evaluate the clinical potential of specific artemisinin compounds in well-designed randomized proof of concept studies. Ultimately, adaptive clinical trials will be required for the identification of the most promising treatment regimens (Uckun, 2020b).

## Discussion

The pharmacokinetics of Artemisinin after a single oral dose was examined in multiple small clinical studies employing Artemisinin most often at the clinically active 500 mg dose level alone or in combination with other antimalarial drugs, such as piperaquine, and showed a rapid elimination within 2–3 h (Duc et al., 1994; De Vries et al., 1997; Ashton et al., 1998; Gordi et al., 2002; Hien et al., 2011; Wang et al., 2020; Li et al., 2021). Due to its time-dependent enzymatic metabolism in the liver by the liver microsomal enzymes CYP2B6 and CYP3A4, the daily systemic exposure level rapidly declines in 5–7 days treatment cycles. This time-dependent pharmacokinetics of Artemisinin and its derivatives have been implicated in the observed high recrudescence rates in malaria patients within 2–3 weeks after monotherapy (Gordi et al., 2002). Therefore, treatment schedules need to be rationally designed for optimal efficacy by taking into consideration both the pathophysiology of target disease, concomitant medications and the pharmacokinetics characteristics of Artemisinin.

High-risk COVID-19 patients have a higher probability of developing a potentially life-threatening multi-system inflammation caused by a cytokine release syndrome (CRS) (Uckun, 2020a; Uckun 2020b; Uckun et al., 2020a; Wu et al., 2020; Zheng et al., 2020; Zhou et al., 2020). Several pro-inflammatory cytokines, including interleukin-6 (IL6), tumor necrosis factor-alpha (TNF-α), and transforming growth factor-beta (TGF-β), contribute to the inflammatory injury of lungs in COVID-19 patients during the CRS (Uckun, 2020b; Uckun et al., 2020b). Notably, infection with SARS-CoV increases the expression of TGF-β and potentiates the TGF-β-regulated MAPK-mediated inflammatory signals (He et al., 2006; Zhao et al., 2008; Li et al., 2016; Wang et al., 2017). These cytokines also contribute to the potentially fatal severe systemic inflammation and multi-organ dysfunction during the viral sepsis of high-risk COVID-19 patients (Uckun, 2020a; Uckun 2020b; Uckun et al., 2020a; Wu et al., 2020; Zheng et al., 2020; Zhou et al., 2020). The reported anti-inflammatory and immunomodulatory effects of Artemisinin and its derivatives have been attributed to their ability to inhibit the pro-inflammatory nuclear factor kappa B (NF-κB) signaling pathway leading to reduced TNF-α and IL-6 levels as well as the Smad2/3-dependent TGF-β signaling pathway (Aldieri et al., 2003; Xu et al., 2007; Wu et al., 2010; He et al., 2011; Mo et al., 2012; Li et al., 2013; Jiang et al., 2016; Zhang et al., 2020). Artemisinin is hoped to mitigate the cytokine-mediated inflammatory injury associated with the cytokine storm and viral sepsis in critically ill COVID-19 patients (Aldieri et al., 2003; Xu et al., 2007; Wu et al., 2010; He et al., 2011; Mo et al., 2012; Li et al., 2013; Jiang et al., 2016; Alhelfawi, 2020; Zhang et al., 2020), in part owing to its ability to block the TGF-β surge which contributes to the development of lung injury and ARDS (Pittet et al., 2001; Budinger et al., 2005; Bossman and Ward, 2014; Frank and Matthay, 2014; Hu and Huang, 2019; Chen, 2020; Zuo et al., 2020). Due to the pivotal role of TGF-β in the pathophysiology of lung fibrosis that develops after an inflammatory injury to the lungs (Xu et al., 2007; Wu et al., 2010; Mo et al., 2012; Wang, 2019; Zhang et al., 2020), the TGF-β pathway inhibitory effect of Artemisinin has the clinical potential to prevent pulmonary fibrosis in COVID-19 patients. It may also help prevent the development of TGF-β triggered serious coagulopathy (Lev et al., 2007; Fox et al., 2020; Stafford et al., 2020). In this regard, data from an ongoing randomized Phase 2 clinical trial of the intravenously administered RNA therapeutic OT101 targeting the TGF-β mRNA that is being conducted in Peru (REPEC (Regsitro Peruano de Ensayos Clinicos):EC INS # PER-067-20) and Argentina (ReNIS (Registro Nacional de Investigaciones en Salud): IS003024) (Uckun et al., 2020b, Uckun and Trieu, 2020). Uzun et al. recently reported that artemisinins might also help reduce the risk of neurologic complications that are encountered in COVID-19 patients (Uzun et al., 2020)

## Conclusion

Artemisinin has a clinical impact potential in the treatment of COVID-19 because it can prevent the progression of the disease and accelerate the recovery of patients before they develop potentially life-threatening complications (Uzun and Toptas, 2020; Krishna et al., 2021). This dual-function COVID-19 drug candidate is hoped to mitigate the cytokine-mediated inflammatory injury associated with the cytokine storm and viral sepsis in critically ill COVID-19 patients.
